# Place and Cause of Death in Centenarians: A Population-Based Observational Study in England, 2001 to 2010

**DOI:** 10.1371/journal.pmed.1001653

**Published:** 2014-06-03

**Authors:** Catherine J. Evans, Yuen Ho, Barbara A. Daveson, Sue Hall, Irene J. Higginson, Wei Gao, on behalf of the GUIDE_Care project

**Affiliations:** 1 Department of Palliative Care, Policy and Rehabilitation, Cicely Saunders Institute, King’s College London, United Kingdom; 2 Sussex Community NHS Trust, Brighton and Hove, United Kingdom; Weill Cornell Medical College, United States of America

## Abstract

**Background:**

Centenarians are a rapidly growing demographic group worldwide, yet their health and social care needs are seldom considered. This study aims to examine trends in place of death and associations for centenarians in England over 10 years to consider policy implications of extreme longevity.

**Methods and Findings:**

This is a population-based observational study using death registration data linked with area-level indices of multiple deprivations for people aged ≥100 years who died 2001 to 2010 in England, compared with those dying at ages 80-99. We used linear regression to examine the time trends in number of deaths and place of death, and Poisson regression to evaluate factors associated with centenarians’ place of death. The cohort totalled 35,867 people with a median age at death of 101 years (range: 100–115 years). Centenarian deaths increased 56% (95% CI 53.8%–57.4%) in 10 years. Most died in a care home with (26.7%, 95% CI 26.3%–27.2%) or without nursing (34.5%, 95% CI 34.0%–35.0%) or in hospital (27.2%, 95% CI 26.7%–27.6%). The proportion of deaths in nursing homes decreased over 10 years (−0.36% annually, 95% CI −0.63% to −0.09%, *p* = 0.014), while hospital deaths changed little (0.25% annually, 95% CI −0.06% to 0.57%, *p* = 0.09). Dying with frailty was common with “old age” stated in 75.6% of death certifications. Centenarians were more likely to die of pneumonia (e.g., 17.7% [95% CI 17.3%–18.1%] versus 6.0% [5.9%–6.0%] for those aged 80–84 years) and old age/frailty (28.1% [27.6%–28.5%] versus 0.9% [0.9%–0.9%] for those aged 80–84 years) and less likely to die of cancer (4.4% [4.2%–4.6%] versus 24.5% [24.6%–25.4%] for those aged 80–84 years) and ischemic heart disease (8.6% [8.3%–8.9%] versus 19.0% [18.9%–19.0%] for those aged 80–84 years) than were younger elderly patients. More care home beds available per 1,000 population were associated with fewer deaths in hospital (PR 0.98, 95% CI 0.98–0.99, *p*<0.001).

**Conclusions:**

Centenarians are more likely to have causes of death certified as pneumonia and frailty and less likely to have causes of death of cancer or ischemic heart disease, compared with younger elderly patients. To reduce reliance on hospital care at the end of life requires recognition of centenarians’ increased likelihood to “acute” decline, notably from pneumonia, and wider provision of anticipatory care to enable people to remain in their usual residence, and increasing care home bed capacity.

*Please see later in the article for the Editors' Summary*

## Abstract 2

Catherine J. Evans and colleagues studied how many and where centenarians in England die, their causes of death, and how these measures have changed from 2001 to 2010.

*Please see later in the article for the Editors' Summary*

## Editors’ Summary

**Background:**

People who live to be more than 100 years old—centenarians—are congratulated and honored in many countries. In the UK, for example, the Queen sends a personal greeting to individuals on their 100th birthday. The number of UK residents who reach this notable milestone is increasing steadily, roughly doubling every 10 years. The latest Office of National Statistics (ONS) figures indicate that 13,350 centenarians were living in the UK in 2012 (20 centenarians per 100,000 people in the population) compared to only 7,740 in 2002. If current trends continue, by 2066 there may be more than half a million centenarians living in the UK. And similar increases in the numbers of centenarians are being seen in many other countries. The exact number of centenarians living worldwide is uncertain but is thought to be around 317,000 and is projected to rise to about 18 million by the end of this century.

**Why Was This Study Done?:**

Traditional blessings often include the wish that the blessing’s recipient lives to be at least 100 years old. However, extreme longevity is associated with increasing frailty—declining physical function, increasing disability, and increasing vulnerability to a poor clinical outcome following, for example, an infection. Consequently, many centenarians require 24-hour per day care in a nursing home or a residential care home. Moreover, although elderly people, including centenarians, generally prefer to die in a home environment rather than a clinical environment, many centenarians end up dying in a hospital. To ensure that centenarians get their preferred end-of-life care, policy makers and clinicians need to know as much as possible about the health and social needs of this specific and unique group of elderly people. In this population-based observational study, the researchers examine trends in the place of death and factors associated with the place of death among centenarians in England over a 10-year period.

**What Did the Researchers Do and Find?:**

The researchers extracted information about the place and cause of death of centenarians in England between 2001 and 2010 from the ONS death registration database, linked these data with area level information on deprivation and care-home bed capacity, and analyzed the data statistically. Over the 10-year study period, 35,867 centenarians (mainly women, average age 101 years) died in England. The annual number of centenarian deaths increased from 2,823 in 2001 to 4,393 in 2010. Overall, three-quarters of centenarian death certificates stated “old age” as the cause of death. About a quarter of centenarians died in the hospital, a quarter died in a nursing home, and a third died in a care home without nursing; only one in ten centenarians died at home. The proportion of deaths in a nursing home increased slightly over the study period but there was little change in the number of hospital deaths. Compared with younger age groups (80–84 year olds), centenarians were more likely to die from pneumonia and “old age” and less likely to die from cancer and heart disease. Among centenarians, dying in the hospital was more likely to be reported to be associated with pneumonia or heart disease than with dementia; death in the hospital was also associated with having four or more contributing causes of death and with living in a deprived area. Finally, living in an area with a higher care-home bed capacity was associated with a lower risk of dying in the hospital.

**What Do These Findings Mean?:**

These findings suggest that many centenarians have outlived death from the chronic diseases that are the common causes of death among younger groups of elderly people and that dying in the hospital is often associated with pneumonia. Overall, these findings suggest that centenarians are a group of people living with a risk of death from increasing frailty that is exacerbated by acute lung infection. The accuracy of these findings is likely to be affected by the quality of UK death certification data. Although this is generally high, the strength of some of the reported associations may be affected, for example, by the tendency of clinicians to record the cause of death in the very elderly as “old age” to provide some comfort to surviving relatives. Importantly, however, these findings suggest that care-home capacity and the provision of anticipatory care should be increased in England (and possibly in other countries) to ensure that more of the growing number of centenarians can end their long lives outside hospital.

**Additional Information:**

Please access these websites via the online version of this summary at http://dx.doi.org/10.1371/journal.pmed.1001653.

- The US National Institute on Aging provides information about healthy aging, including information on longevity (in English and Spanish)
- The National End of Life Care Intelligence Network, England is a government organization that gathers data on care provided to adults approaching the end of life to improve service quality and productivity
- The Worldwide Palliative Care Alliance promotes universal access to affordable palliative care through the support of regional and national palliative care organizations
- The non-for-profit organization AgeUK provides information about all aspects of aging
- Wikipedia has a page on centenarians (note that Wikipedia is a free online encyclopedia that anyone can edit; available in several languages)
- The International Longevity Centre-UK is an independent, non-partisan think tank dedicated to addressing issues of longevity, ageing and population; its “Living Beyond 100” report examines the research base on centenarians and calls for policy to reflect the ongoing UK increase in extreme longevity
- This study is part of GUIDE_Care, a project initiated by the Cicely Saunders Institute to investigate patterns in place of death and the factors that affect these patterns

## Introduction

People aged 100 years or over are a rapidly growing demographic group worldwide. In 2011, centenarians globally numbered 317,000. They are projected to grow to 3,224,000 by 2050, reaching 17,795,000 at the end of the century [1]. In the UK, this group has steadily increased since 1956 with numbers roughly doubling every 10 years [2],[3] and estimated to reach over half a million by 2066 [4]. Throughout Europe women centenarians outnumber men, but with evidence of levelling with comparative gains in male life expectancy [2].

The risk of requiring a care home placement increases with advancing age. In the US, 58% of people aged over 95 years die in a care home; 28% die in hospital [5]. In contrast in the UK, although 51% aged over 90 years require long-term care at the end of life (EoL) (e.g., a care home 40%) [6], only 38% die in a care home while 52% die in hospital [7]. The proportion of hospital deaths in advanced age is similar to the whole population (50%) [8]. A main driver of the cost of end of life care (EoLC) is hospital admission in the last weeks of life, accounting for 33% of the mean total cost per patient [9]. Older people’s preference for place of death is outside of hospital [10] in a “homely” environment characterised by familiarity, autonomy, and presence of loved ones. “Homeliness” is associated with the attributes of the setting and may be a communal facility (e.g., a care home), and not limited to one’s own residence [11]. The EoLC Strategy for England advocates improving EoLC across all care settings, notably care homes, by enhancing the timeliness, responsiveness, and co-ordination of care [12].

Few studies consider the explicit health and social care needs of centenarians [13],[14] compared to younger cohorts of older people or the implications of extreme longevity for policy and service delivery [15]. An important factor in informing EoLC policy and practice is examination of cause of death data as a predictor of place of death using for example death registration data [16]. However, analysing data on centenarians is relatively uncommon. Most studies do not consider those aged 100 years or over as a separate cohort even though their longevity is remarkable. National and international studies have analysed death registration data to inform ageing strategies for the oldest old, but seldom differentiate centenarians within this group [6],[7],[17]–[19] or are disease specific, for example, cancer [20].

No study to our knowledge has considered trends in place of death and associations for centenarians. The increasing trend of extreme longevity for men and women requires detailed investigation to inform policy and service provision [15]. Death registration data in the UK is considered of sufficiently high quality to support policy development and implementation [21]. This study aims to examine trends in place of death and associations for centenarians in England over 10 years to consider implications of extreme longevity for health and social care and variation with cohorts aged 80 to 99 years.

## Methods

We carried out a population-based observational study (Checklist S1) [22].

### Ethics Statement

Following Office for National Statistics (ONS) procedures a Data Access Agreement was signed detailing data management and protection, and Individual Approvals granted after assessment of researchers accessing the ONS data (YH, WG, and IJH). The study used anonymous records and no ethical approval was required in accordance with the Information Commissioner’s Office guidelines, ONS procedures, and King’s College London Research Ethics Committee.

### Data Sources

We used ONS death registration data for England 2001–2010. The database details decedents’ age, gender, marital status, usual residence, place of death and year of death, underlying cause of death, and contributing causes of death (up to 15) using *International Classification of Diseases Tenth Revision* (ICD-10) [23].

We linked the ONS death registration database with area level data on: deprivation, settlement type of place of residence, and care home bed capacity. The linkage with index of multiple deprivation indices (IMD) 2010 [24] was based on Lower Super Output Area (LSOA) of the decedents’ usual residence. The IMD 2010 is a composite measure of deprivation used at the LSOA level and summarised as quintiles based on national rankings [24]. Settlement type (e.g., urban, town, or village) was generated from usual residential address at LSOA level. Data linkage between ONS place of residence with data from the Care Quality Commission (http://www.cqc.org.uk) identified the number of care home beds (nursing home and residential care homes) per 1,000 population by decedents’ local authority district.

### Inclusion Criteria

Inclusion criteria comprised individuals aged ≥100 years at time of death and who died in England between 2001 and 2010 (inclusive) from all causes of death, excluding external causes of accident or violence [23]. For comparison, data from the same timeframe using the same exclusion criteria were included for those who died age 80–99.

### Main Outcome

The place of death was grouped into five categories: hospital, nursing home, residential care home, at home, or elsewhere. Care homes provide 24-hour long-term care, categorised in the UK as with nursing (nursing home) or without (residential care home) [25]. Care homes without nursing provide personal care and residents’ health needs and access to specialist services are served by primary health care services, notably general practitioners (physicians in primary care) and community nurses [26],[27].

### Explanatory Variables

We examined factors associated with place of death [28]. Explanatory variables were grouped as individual level data: (1) demographic factors (age, gender and marital status, usual residence); (2) illness related (ICD-10 codes for the top eight underlying causes of death and contributing causes of death); and regional level (3) environmental (deprivation, settlement type [e.g., rural, urban], number of care homes per 1,000 population). Detail of usual residence of a decedent is supplied by the informant to the registrar. Since 1993 the informant decides which address to give if more than one is applicable. For example, an informant may consider that the deceased was not resident in a communal establishment (e.g., a care home) where the death occurred and provides a private address to the registrar even though the deceased had lived in the communal establishment for several months [23].

### Data Analysis

We used simple linear regression to analyse trends in centenarians’ place of death from 2001 to 2010, place of death and cause of death, and descriptive analysis to explore demographic characteristics, causes of death, contributing causes, and environmental factors (e.g., deprivation indices, died in usual residence). Causes of death were classified as: prominent specific disease types (e.g., pneumonia ICD-10 J12-J118), or disease group (e.g., other respiratory ICD-10 J [others]). Uncommon causes of death were collapsed into “other” (those identified as outside the prominent ICD-10 codes). Prominent groups were entered into the bivariate analysis using frequency tables and descriptive statistics (e.g., proportions and 95% confidence intervals) to explore place of death and variation by gender, marital status, causes of death, number of contributing causes, deprivation, region, and settlement (urban/rural). Findings informed candidate variables for regression modelling on associations with place of death with ten candidate variables grouped as individual level data: (1) demographic; (2) illness; and (3) regional level environmental data. We used multivariable Poisson regression with robust error variance to calculate proportional ratios (PRs) [29], to investigate factors associated with hospital death versus care home (nursing home or residential home), or at home. Age remained in the model as a continuous variable. We report *p*-values and confidence intervals to enable inferences to centenarian populations outside England and to future centenarian cohorts. We checked residuals to test model specification [30]. We used descriptive analysis to compare cause of death and place of death by age from 80 to ≥100 divided into 5 year age bands. All analysis was undertaken using R version 2.15.1 [31].

## Results

The number of centenarian deaths per year in England increased by 56% (95% CI 53.8%–57.4%) in 10 years from 2,823 in 2001 to 4,393 in 2010. The 10 year cohort comprised 35,867 people with a median age of 101 years (range: 100–115 years) at time of death, who were mainly women (86.7%) and widowed (85.0%) (Table 1). Areas of highest deprivation had the lowest proportion of centenarian deaths with consequent regional variance by level of deprivation. The north east of England had the lowest proportion of centenarian deaths (4.3%) (Table 1).

**Table 1:** Demographic characteristics of all centenarian deaths in England 2001–2010.

Characteristic	Subgroup	Number	Percent
All	Total deaths	35,867	—
Age	Mean (SD)	101.4 (1.7)	—
	Median (min–max)	101 (100–115)	—
Gender	Women	31,096	86.7%
	Men	4,771	13.3%
Marital status	Widowed	30,397	84.7%
	Single	4,041	11.3%
	Divorced	571	1.6%
	Married	749	2.1%
	Unknown	109	0.3%
Number contributing causes	0	15,220	42.4%
	1	12,939	36.1%
	2	5,221	14.6%
	3	1,701	4.7%
	4+	786	2.2%
Died in usual residential place	Yes	18,346	51.2%
	No	17,521	48.8%
IMD 2010	1 (Least deprived)	7,259	20.2%
	2	8,503	23.7%
	3	8,328	23.2%
	4	6,721	18.7%
	5 (Most deprived)	5,056	14.1%
Region^a^	North East	1,557	4.3%
	North West	4,374	12.2%
	Yorkshire and the Humber	3,526	9.8%
	East Midlands	2,921	8.1%
	West Midlands	3,319	9.3%
	East of England	4,099	11.4%
	London	3,878	10.8%
	South East Coast	4,442	12.4%
	South Central	2,683	7.5%
	South West	5,068	14.1%
Settlement type	Urban	27,820	77.6%
	Town and fringe	4,199	11.7%
	Village, hamlet, and isolated dwelling	3,848	10.7%
Place of death	Hospital	9,740	27.2%
	Nursing home	9,581	26.7%
	Residential home	12,369	34.5%
	Own home	3,460	9.6%
	Hospices	74	0.2%
	Others	643	1.8%

a The region was defined by Strategic Health Authorities (SHAs) (July 2006) [43].

SD, standard deviation.

**Figure 1:**
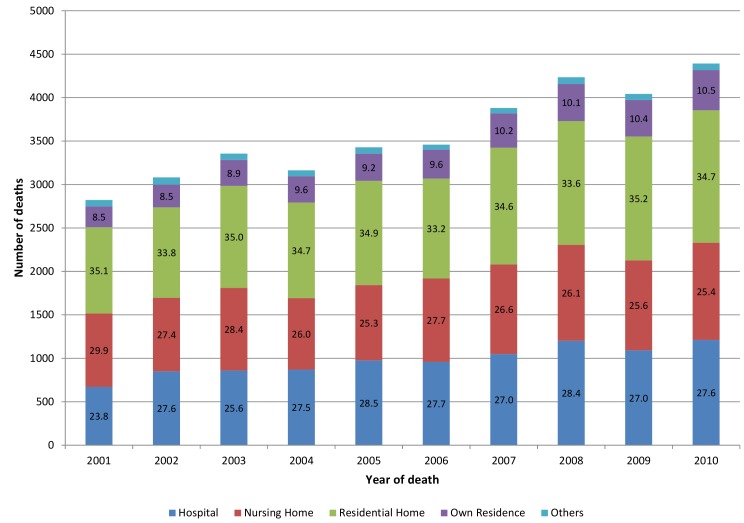
Centenarian deaths by place of death 2001–2010 (*n*, %). Bar number  = % proportion.

Most centenarians died in a residential care home (34.5%, 95% CI 34.0%–35.0%) or nursing home (26.7%, 95% CI 26.3%–27.2%); few died at home (9.6%, 95% CI 9.3%–10.0%) or in a hospice (0.2%, 95% CI 0.2%–0.3%). Over a quarter (27.2%, 95% CI 26.7%–27.6%) died in hospital (Table 1). Nearly half died outside their usual address (48.8%, 95% CI 48.3%–49.4%). Trends in place of death significantly changed in two places: nursing homes decreased (−0.36% annually, 95% CI −0.63% to −0.09%, *p* = 0.014), but changes in raw numbers were small (*n* = 845 in 2001; *n* = 1,118 in 2010); and own home increased (0.24% annually, 95% CI 0.18%–0.29%, *p*<0.001), but the raw numbers were small (*n* = 241 in 2001 to *n* = 463 in 2010) (Figure 1). Little variation was evident in the proportion dying in hospital (0.25% annually, 95% CI −0.06% to 0.57%, *p* = 0.09) or residential care homes (−0.01% annually, 95% CI −0.19% to 0.17%, *p* = 0.88).

Seven disease groups classified by ICD-10 disease groups accounted for 90.2% (95% CI 89.9%–90.5%) of centenarians’ underlying cause of death with the remainder classified as “other” (9.8%, 95% CI 9.5%–10.1%) (Table 2). The prominent causes of death comprised: pneumonia (17.7%, 95% CI 17.3%–18.1%) and other respiratory diseases (6.0%, 95% CI 8.3%–8.9%); cerebrovascular (10.0%, 95% CI 9.7%–10.3%); ischaemic heart diseases (8.6%, 95% CI 8.3%–8.9%) and other circulatory diseases (9.8%, 95% CI 9.5%–10.1%); dementia and Alzheimer disease (5.7%, 95% CI 5.4%–5.9%); cancer (4.4%, 95% CI 4.2%–4.6%); senility “old age” (28.1%, 95% CI 4.2%–4.6%). “Old age” formed the largest ICD-10 grouping (28.1%, 95% CI 27.6%–28.5%) (Table 2). However, trends in certifying death as “old age” showed a decreasing trend over 10 years, notably in hospital (−0.87% annually, 95% CI −1.09 to −0.65%, *p*<0.001) with increasing use of dementia (0.34% annually, 95% CI 0.14%–0.54%, *p* = 0.004).

**Table 2:** Centenarian cause of death by place of death in England 2001–2010.

Among Deaths in PoD, How Many of Them Died with CoD?		PoD											
ICD-10 Code	CoD	Hospital*n* = 9,740		Nursing Home*n* = 9,581		Residential Home*n* = 12,369		Own Home*n* = 3,460		Others*n* = 717		All*n* = 35,867	
		Percent	(95% CI)	Percent	(95% CI)	Percent	(95% CI)	Percent	(95% CI)	Percent	(95% CI)	Percent	(95% CI)
(J12–J18)	Pneumonia	21.8	(21.0%–22.6%)	16.9	(16.2%–17.7%)	16.6	(15.9%–17.2%)	13.7	(12.5%–14.8%)	13.4	(10.9%–15.9%)	17.7	(17.3%–18.1%)
(I60–I69)	Cerebro–vascular	12.4	(11.8%–13.1%)	9.9	(9.3%–10.5%)	9.1	(8.6%–9.6%)	6.6	(5.8%–7.4%)	9.3	(7.2%–11.5%)	10.0	(9.7%–10.3%)
(I [others])	Circulatory others	11.4	(10.7%–12.0%)	8.9	(8.3%–9.4%)	8.9	(8.4%–9.4%)	11.0	(10%–12.1%)	10.6	(8.3%–12.9%)	9.8	(9.5%–10.1%)
(I20–I25)	Ischaemic heart disease	11.9	(11.2%–12.5%)	6.3	(5.8%–6.7%)	7.0	(6.5%–7.4%)	11.8	(10.7%–12.9%)	8.4	(6.3%–10.4%)	8.6	(8.3%–8.9%)
(J [others])	Respiratory others	6.9	(6.4%–7.4%)	5.6	(5.1%–6.0%)	5.7	(5.3%–6.1%)	5.4	(4.7%–6.2%)	6.8	(5%–8.7%)	6.0	(5.7%–6.2%)
(F01–F03 and G30)	Dementia	3.1	(2.8%–3.5%)	8.6	(8.0%–9.1%)	6.3	(5.9%–6.7%)	2.7	(2.2%–3.3%)	5.9	(4.1%–7.6%)	5.7	(5.4%–5.9%)
(C)	Cancer	4.5	(4.1%–4.9%)	3.7	(3.3%–4.1%)	4.0	(3.6%–4.3%)	6.0	(5.2%–6.8%)	12.0	(9.6%–14.4%)	4.4	(4.2%–4.6%)
(R53–R54)	“Old age”	9.7	(9.1%–10.2%)	34.2	(33.2%–35.1%)	35.9	(35.1%–36.8%)	35.5	(33.9%–37.1%)	24.1	(21%–27.3%)	28.1	(27.6%–28.5%)
Excluding above codes	“Others”	18.3	(17.5%–19.1%)	6.1	(5.6%–6.5%)	6.6	(6.2%–7.1%)	7.3	(6.4%–8.1%)	9.5	(7.3%–11.6%)	9.8	(9.5%–10.1%)

CoD, cause of death; PoD, place of death.

The main causes of death varied by place of death. Pneumonia (21.8%, 95% CI 21.0%–22.6%) accounted for the largest group of hospital deaths, while across non-hospital settings “old age” formed the largest category and then pneumonia (Table 2). Cancer accounted for a small proportion of deaths across care settings, except in “other” settings (including hospices), accounting for 12.0% (95% CI 9.6%–14.4%) of deaths. Dementia accounted for few deaths either as an underlying cause (5.7%, 95% CI 5.4%–5.9%) or as a contributing cause (4.4%, 95% CI 4.2%–4.7%). Overall, recording multiple contributing causes of death was uncommon; most individuals had none (42.4%, 95% CI 41.9%–42.9%) or one (36.1%, 95% CI 35.6%–36.6%) (Table 1). Commonest contributing causes were: circulatory (12.1%, 95% CI 11.7%–12.5%), pneumonia (12.0%, 95% CI 11.6%–12.4%), “others” (9.0%, 95% CI 8.7%–9.4%), and dementia (4.4%, 95% CI 4.2%–4.7%). “Old age” formed the largest group (47.5%, 95% CI 46.9%–48.1%). Overall, 75.6% of centenarian death certifications stated “old age” as either an underlying cause (28.1%) or contributing cause (47.5%).

The main causes of death changed with increasing age (Table 3). Centenarians had relatively lower rates of chronic diseases as causes of death compared to the younger cohorts. In the youngest cohort aged 80–85 years ischaemic heart disease accounted for 19.0% (95% CI 18.9%–19.0%) of death certifications, compared to 8.6% (95% CI 8.3%–8.9%) for centenarians. Centenarians were certified as dying more often from pneumonia (17.7%, 95% CI 17.3–18.1) and “old age” (28.1%, 95% 27.7%–28.5%), compared to the youngest cohort (pneumonia 6%, 95% CI 5.9%–6.0%; “old age” 0.9%, 95% CI 0.9%–0.9%). Cause of death and place of death changed markedly in extreme old age compared to the “younger” age bands. Death from pneumonia in hospital increased 3-fold for centenarians to 21.8% (95% CI 21.0%–22.6%) from 7.3% (95% CI 7.2%–7.3%) for those aged 80–84 years (Table 4). Correspondingly, common causes of death in hospital prevalent in “younger” age groups declined in extreme old age, notably Ischemic heart disease and cancer (Table 4). Dying outside of hospital from “old age” increased markedly with advancing age. Over a third (34.2%, 95% CI 33.2%–35.1%) of centenarian deaths in nursing homes were certified as “old age” compared to only 2.4% (95% CI 2.3%–2.5%) for 80–84 year olds (Table 5). Dementia as cause of death decreased with advancing age in settings outside of hospital (Tables 5–8).

**Table 3:** All causes of death by age group.

CoD	Age Group									
	80–84 Years*n* = 848,674		85–89 Years*n* = 804,740		90–94 Years*n* = 533,216		95–99 Years*n* = 203,230		100+ Years*n* = 35,867	
	Percent	(95% CI)	Percent	(95% CI)	Percent	(95% CI)	Percent	(95% CI)	Percent	(95% CI)
Pneumonia	6.0	5.9–6.0	8.3	8.2–8.3	11.5	11.4–11.6	14.6	14.5–14.8	17.7	17.3–18.1
Cerebro-vascular	12.1	12.0–12.1	13.9	13.8–13.9	14.5	14.4–14.6	13.2	13.1–13.4	10.0	9.7–10.3
Circulatory others	9.8	9.7–9.9	10.8	10.7–10.8	11.3	11.2–11.3	11.1	10.9–11.2	9.8	9.5–10.1
Ischaemic heart disease	19.0	18.9–19.0	17.8	17.7–17.9	15.5	15.4–15.6	12.8	12.7–13.0	8.6	8.3–8.9
Respiratory others	4.6	4.5–4.6	6.6	6.5–6.6	8.0	7.9–8.0	8.4	8.2–8.5	5.7	5.4–5.9
Dementia	9.4	9.3–9.5	8.4	8.3–8.5	7.2	7.2–7.3	6.7	6.6–6.8	6.0	5.7–6.2
Cancer	24.5	24.6-25.4	17.8	17.7–17.9	11.9	11.8–12.0	7.8	7.7–8.0	4.4	4.2–4.6
“Old age”	0.9	0.9–0.9	2.3	2.2–2.3	6.2	6.1–6.3	12.5	12.4–12.6	28.1	27.6–28.5
“Others”	13.8	13.9–14.0	14.2	14.2–14.3	13.9	13.8–14.0	12.9	12.7–13.0	9.8	9.5–10.1

CoD, cause of death.

**Table 4:** Cause of death by age bands and hospital as place of death.

CoD	Hospital PoD by 5 Year Age Bands									
	80–84 years*n* = 496,888		85– 89 years*n* = 452,161		90–94 years*n* = 268,059		95–99 years*n* = 84,324		100+ years*n* = 9,740	
	Percent	(95% CI)	Percent	(95% CI)	Percent	(95% CI)	Percent	(95% CI)	Percent	(95% CI)
Pneumonia	7.3	7.2–7.3	9.7	9.6–9.8	13.1	13.0–13.3	16.5	16.3–16.8	21.8	21.0–22.6
Cerebro-vascular	13.0	12.9–13.1	14.2	14.1–14.3	14.8	14.7–14.9	14.2	13.9–14.4	12.4	11.8–13.1
Circulatory	10.6	10.5–10.7	11.5	11.4–11.6	11.9	11.8–12.1	11.9	11.6–12.1	11.4	10.7–12.0
IHD	18.5	18.4–18.7	18.1	18.0–18.2	16.6	16.5–16.8	15.1	14.8–15.3	11.9	11.2–12.5
Respiratory (others)	10.9	10.8–10.9	9.6	9.5–9.7	8.2	8.1–8.3	7.7	7.5–7.9	6.9	6.4–7.4
Dementia and AD	2.5	2.5–2.6	3.6	3.5–3.6	4.3	4.3–4.4	4.7	4.5–4.8	3.1	2.8–3.5
Cancer	19.6	19.4–19.7	14.5	14.4–14.6	10.2	10.1–10.3	6.9	6.7–7.1	4.5	4.1–4.9
“Old age”/frailty	0.4	0.3–0.4	0.8	0.8–0.8	2.1	2.1–2.2	4.1	3.9–4.2	9.7	9.1–10.2
Other	17.3	17.1–17.4	18.0	17.9–18.1	18.6	18.5–18.8	19.1	18.8–19.3	18.3	17.5–19.1

AD, Alzheimer disease; CoD, cause of death; IHD, ischaemic heart disease; PoD, place of death.

**Table 5:** Cause of death by age bands and nursing home as place of death.

CoD	Nursing Home PoD by Five Year Age Bands									
	80–84 years*n* = 96,687		85–89 years*n* = 122,627		90–94 years*n* = 102,871		95–99 years*n* = 47,241		100+ years*n* = 9,581	
	Percent	(95% CI)	Percent	(95% CI)	Percent	(95% CI)	Percent	(95% CI)	Percent	(95% CI)
Pneumonia	6.0	5.8–6.1	7.9	7.7–8.0	10.7	10.5–10.9	13.6	13.3–13.9	16.9	16.2–17.7
Cerebro-vascular	19.8	19.5–20.0	19.7	19.5–19.9	17.9	17.7–18.1	14.8	14.5–15.2	9.9	9.3–10.5
Circulatory	6.3	6.2–6.5	7.7	7.6–7.9	8.9	8.8–9.1	9.5	9.2–9.7	8.9	8.3–9.4
IHD	8.9	8.7–9.1	9.8	9.6–9.9	9.8	9.6–9.9	8.7	8.5–9.0	6.3	5.8–6.7
Respiratory (others)	7.5	7.4–7.7	6.9	6.8–7.0	6.3	6.2–6.5	6.2	5.9–6.4	5.6	5.1–6.0
Dementia and AD	14.8	14.6–15.1	15.7	15.5–15.9	15.0	14.8–15.2	13.2	12.9–13.5	8.6	8.0–9.1
Cancer	21.3	21.1–21.6	16.3	16.1–16.5	11.2	11.0–11.4	7.4	7.2–7.6	3.7	3.3–4.1
“Old age”/frailty	2.4	2.3–2.5	4.8	4.7–5.0	10.4	10.2–10.6	18.0	17.6–18.3	34.2	33.2–35.1
Other	12.8	12.6–13.0	11.1	11.0–11.3	9.6	9.5–9.8	8.6	8.3–8.8	6.1	5.6–6.5

AD, Alzheimer disease; CoD, cause of death; IHD, ischaemic heart disease; PoD, place of death.

**Table 6:** Cause of death by five year age band and residential home as place of death.

CoD	Residential Care Home PoD by Five Year Age Bands									
	80–84 Years*n* = 55,916		85–89 Years*n* = 86,325		90–94 Years*n* = 89,742		95–99 Years*n* = 48,610		100+ Years*n* = 12,369	
	Percent	(95% CI)	Percent	(95% CI)	Percent	(95% CI)	Percent	(95% CI)	Percent	(95% CI)
Pneumonia	6.9	6.7–7.1	8.9	8.7–9.0	11.6	11.4–11.8	14.3	14.0–14.6	16.6	15.9–17.2
Cerebro-vascular	17.0	16.7–17.3	16.4	16.2–16.7	14.8	14.5–15.0	12.3	12.0–12.6	9.1	8.6–9.6
Circulatory	7.7	7.5–7.9	8.9	8.7–9.1	9.9	9.7–10.1	9.9	9.7–10.2	8.9	8.4–9.4
IHD	12.7	12.5–13.0	12.7	12.5–12.9	12.0	11.8–12.2	10.2	9.9–10.4	7.0	6.5–7.4
Respiratory (others)	8.0	7.8–8.2	7.4	7.2–7.6	6.4	6.2–6.6	6.0	5.7–6.2	5.7	5.3–6.1
Dementia and AD	15.7	15.4–16.0	15.9	15.7–16.2	14.0	13.8–14.3	11.8	11.5–12.1	6.3	5.9–6.7
Cancer	16.8	16.5–17.1	13.1	12.9–13.3	9.5	9.3–9.7	6.9	6.7–7.1	4.0	3.6–4.3
“Old age”/frailty	3.2	3.1–3.4	6.1	6.0–6.3	12.4	12.2–12.6	20.3	19.9–20.7	35.9	35.1–36.8
Other	12.0	11.7–12.2	10.5	10.3–10.7	9.5	9.3–9.7	8.4	8.1–8.6	6.6	6.2–7.1

AD, Alzheimer disease; CoD, cause of death; IHD, ischaemic heart disease; PoD, place of death.

**Table 7:** Cause of death by age bands and own home as place of death.

CoD	Own Home PoD by Five Year Age Bands									
	80–84 Years*n* = 145,460		85–89 Years*n* = 106,564		90–94 Years*n* = 54,687		95–99 Years*n* = 17,961		100+ Years*n* = 3,460	
	Percent	(95% CI)	Percent	(95% CI)	Percent	(95% CI)	Percent	(95% CI)	Percent	(95% CI)
Pneumonia	2.8	2.7–2.8	4.0	3.8–4.1	6.8	6.6–7.0	10.4	9.9–10.8	13.7	12.5–14.8
Cerebro-vascular	4.4	4.3–4.5	5.9	5.7–6.0	7.3	7.1–7.5	8.0	7.6–8.4	6.6	5.8–7.4
Circulatory	12.1	11.9–12.3	14.1	13.9–14.3	15.3	15.0–15.7	14.6	14.1–15.2	11.0	10.0–12.1
IHD	32.8	32.6–33.0	32.3	32.0–32.6	27.6	27.2–28.0	20.2	19.6–20.8	11.8	10.7–12.9
Respiratory (others)	8.2	8.0–8.3	7.3	7.2–7.5	6.2	6.0–6.4	5.7	5.4–6.1	5.4	4.7–6.2
Dementia and AD	1.3	1.2–1.3	2.2	2.1–2.3	3.2	3.0–3.3	3.7	3.4–4.0	2.7	2.2–3.3
Cancer	30.7	30.5–31.0	24.3	24.0–24.5	16.9	16.6–17.2	11.6	11.1–12.1	6.0	5.2–6.8
“Old age”/frailty	0.9	0.8–0.9	2.8	2.7–2.8	8.7	8.5–9.0	17.6	17.1–18.2	35.5	33.9–37.1
Other	6.8	6.7–7.0	7.2	7.1–7.4	7.9	7.7–8.1	8.2	7.8–8.6	7.3	6.4–8.1

AD, Alzheimer disease; CoD, cause of death; IHD, ischaemic heart disease; PoD, place of death.

**Table 8:** Cause of death by five year age band and “other” place of death.

CoD	“Other” PoD by Five Year Age Bands									
	80–84 Years*n* = 53,723		85–89 Years*n* = 37,063		90–94 Years*n* = 17,857		95–99 Years*n* = 5,094		100+ Years*n* = 717	
	Percent	(95% CI)	Percent	(95% CI)	Percent	(95% CI)	Percent	(95% CI)	Percent	(95% CI)
Pneumonia	1.6	1.5–1.7	3.2	3.0–3.4	6.4	6.0–6.7	10.1	9.2–10.9	13.4	10.9–15.9
Cerebro-vascular	4.8	4.6–5.0	7.2	6.9–7.5	10.6	10.2–11.1	11.1	10.2–11.9	9.3	7.2–11.5
Circulatory	4.3	4.1–4.4	6.2	5.9–6.4	8.6	8.2–9.0	11.4	10.5–12.2	10.6	8.3–12.9
IHD	9.9	9.6–10.1	11.5	11.1–11.8	12.8	12.3–13.3	11.9	11.0–12.8	8.4	6.3–10.4
Respiratory (Others)	3.8	3.6–4.0	4.4	4.2–4.6	4.8	4.5–5.2	5.1	4.5–5.7	6.8	5.0–8.7
Dementia and AD	2.5	2.3–2.6	4.1	3.9–4.3	6.1	5.8–6.5	7.9	7.1–8.6	5.9	4.1–7.6
Cancer	67.1	66.7–67.5	55.2	54.7–55.7	37.5	36.8–38.3	22.9	21.8–24.1	12.0	9.6–14.4
“Old age”/frailty	0.3	0.3–0.4	1.2	1.1–1.3	4.3	4.0–4.6	9.4	8.6–10.2	24.1	21.0–27.3
Other	5.7	5.5–5.9	7.1	6.9–7.4	8.8	8.4–9.2	10.3	9.5–11.1	9.5	7.3–11.6

AD, Alzheimer disease; CoD, cause of death; IHD, ischaemic heart disease; PoD, place of death.

### Factors Associated with Centenarians’ Place of Death

#### Demographic factors

Women were more likely to die outside of hospital in a community setting of a care home (with or without nursing) and own residence, compared with men (Table 9). Marital status was not associated with place of death (Table 9).

**Table 9:** Proportion ratios and 95% CI of variables associated with place of death (hospital reference group) in England 2001–2010.

Variable	Value	Hospital Versus Nursing Home			Hospital Versus Residential Home			Hospital Versus Own Home		
		PR	95% CI	*p*-Value	PR	95% CI	*p*-Value	PR	95% CI	*p*-Value
Age		0.98	(0.97–0.99)	<0.001	0.96	(0.95–0.97)	<0.001	0.98	(0.97–0.99)	<0.001
Gender	Women	1.00		<0.001			<0.001			<0.001
	Men	1.26	(1.22–1.30)		1.33	(1.28–1.37)		1.07	(1.04–1.09)	
Marital Status	Widowed	1.00		0.08			0.015			0.36
	Single	0.91	(0.86–0.95)		0.89	(0.85–0.94)		1.03	(1.00–1.06)	
	Divorced	0.96	(0.87–1.07)		0.94	(0.84–1.05)		0.94	(0.87–1.02)	
	Married	1.13	(1.07–1.20)		1.19	(1.12–1.27)		0.88	(0.83–0.93)	
Underlying cause of death	Dementia (F01–F03, G30)	1.00		<0.001			<0.001			<0.001
(ICD10 codes)	Pneumonia (J12–J18)	1.97	(1.78–2.17)		1.69	(1.53–1.86)		1.04	(0.98–1.10)	
	Cerebrovascular (I60–I69)	1.97	(1.78–2.18)		1.75	(1.58–1.93)		1.06	(1.00–1.13)	
	Circulatory others (I [others])	1.92	(1.74–2.13)		1.61	(1.46–1.78)		0.93	(0.88–0.99)	
	Ischaemic heart disease (I20–I25)	2.19	(1.98–2.42)		1.81	(1.64–2.00)		0.92	(0.86–0.97)	
	Respiratory others (J [others])	1.90	(1.70–2.11)		1.57	(1.41–1.75)		0.98	(0.92–1.05)	
	Cancer (C)	1.87	(1.67–2.09)		1.51	(1.35–1.69)		0.86	(0.79–0.92)	
	“Old age” (R53–R54)	0.78	(0.69–0.87)		0.58	(0.52–0.65)		0.55	(0.51–0.59)	
	“Others”	2.45	(2.23–2.70)		2.11	(1.91–2.32)		1.08	(1.02–1.15)	
Contributing causes of death	0	1.00		<0.001			<0.001			<0.001
	1	0.84	(0.81–0.87)		0.80	(0.77–0.84)		0.91	(0.88–0.93)	
	2	1.01	(0.97–1.06)		1.01	(0.97–1.05)		1.01	(0.99–1.04)	
	3	1.13	(1.08–1.19)		1.19	(1.13–1.25)		1.08	(1.05–1.12)	
	4+	1.27	(1.21–1.33)		1.36	(1.29–1.43)		1.15	(1.11–1.19)	
Index of multiple deprivation	1 (least deprived)	1.00		0.016			0.02			0.79
	2	1.01	(0.97–1.05)		1.01	(0.96–1.05)		1.01	(0.98–1.05)	
	3	1.03	(0.99–1.07)		0.97	(0.93–1.02)		1.01	(0.98–1.04)	
	4	1.10	(1.06–1.15)		1.04	(1.00–1.09)		1.05	(1.01–1.08)	
	5 (most deprived)	1.16	(1.11–1.21)		1.13	(1.08–1.19)		1.05	(1.02–1.09)	
Region	North East	1.00		<0.001			<0.001			0.924
	North West	0.95	(0.88–1.02)		0.97	(0.90–1.05)		0.98	(0.93–1.03)	
	Yorkshire and the Humber	0.94	(0.87–1.02)		0.95	(0.88–1.03)		0.95	(0.90–1.00)	
	East Midlands	0.93	(0.85–1.00)		0.89	(0.81–0.96)		0.97	(0.92–1.02)	
	West Midlands	1.00	(0.92–1.07)		0.95	(0.88–1.03)		0.94	(0.89–0.99)	
	East of England	1.13	(1.05–1.22)		0.89	(0.82–0.96)		0.91	(0.86–0.96)	
	London	1.19	(1.11–1.28)		1.16	(1.08–1.25)		0.96	(0.92–1.01)	
	South East Coast	0.99	(0.92–1.07)		0.98	(0.91–1.06)		0.94	(0.90–0.99)	
	South Central	1.08	(1.00–1.17)		0.87	(0.80–0.95)		0.92	(0.86–0.97)	
	South West	0.92	(0.85–0.99)		0.86	(0.79–0.93)		0.90	(0.86–0.95)	
Settlement type	Urban	1.00		0.10			0.05			0.008
	Town and fringe	1.04	(0.99–1.08)		0.89	(0.85–0.94)		0.95	(0.92–0.99)	
	Village, hamlet and isolated dwelling	0.90	(0.86–0.95)		0.95	(0.90–1.00)		0.85	(0.81–0.88)	
Number of care home beds per 1,000 population		0.98	(0.98–0.99)	<0.001	0.98	(0.97–0.98)	<0.001	1.00	(1.00–1.01)	0.008

A PR greater than 1 indicates higher probability of death in hospital.

#### Illness factors

Underlying causes of death (reference group: dementia) were associated with place of death (*p*<0.001; Table 9). Compared to people with dementia, people with an underlying cause of death from cancer (PR 0.86, 95% CI 0.79–0.92), ischaemic heart disease (PR 0.92, 95% CI 0.86–0.97), or other circulatory diseases (PR 0.93, 95% CI 0.88–0.99) were less likely to die in hospital than at home (Table 9). Only “old age” as a cause of death compared to people dying from dementia was associated with being less likely to die in hospital compared with a nursing home (PR 0.78, 95% CI 0.69–0.87) or residential care home (PR 0.58, 95% CI 0.52–0.65, *p*<0.001). Dying with a more cumulative picture of disease with certification of ≥4 contributing causes of death was associated with dying in hospital rather than a care home, either with nursing (PR 1.27, 95% CI 1.21–1.33) or without (PR 1.36, 95% CI 1.29–1.43). Conversely, those dying with a single contributing cause were less likely to die in hospital and more likely to die in a care home with nursing (PR 0.84, 95% CI 0.81–0.87) or without (PR 0.80, 95% CI 0.77–0.84), or in their own residence (PR 0.91, 95% CI 0.88–0.93).

#### Environmental factors

Higher numbers of care home beds were associated with fewer hospital deaths and more deaths in care homes (with nursing PR 0.98, 95% CI 0.98–0.99, *p*<0.001; or without nursing PR 0.98, 95% CI 0.97–0.98, *p*<0.001). The main difference between dying in hospital and care home type pertained to a higher prevalence of dementia in nursing homes (21.3% versus 16.5%) (Table 9). Once removed from the model, minimal differences were observed between cause of death and care home type (Chi^2^ 13.98, degrees of freedom (df)  = 7, *p* = 0.051).

Place of death was associated with level of deprivation and settlement type. Areas most deprived showed greatest association with dying in hospital rather than in a community setting (Table 9). Dying outside of hospital in one’s own residence related to usual residence of urban versus rural settlement (PR 0.85, 95% CI 0.81–0.89) or town/fringe (PR 0.95, 95% CI 0.92–0.99).

## Discussion

This is the first study to our knowledge to examine trends in place of death for centenarians and the associated factors. Centenarians are a group who have outlived chronic diseases common as causes of death amongst “younger” older cohorts. Centenarians are a group whose death is often certified as from frailty/”old age” and pneumonia. Over three-quarters of death certifications stated “old age” as either an underlying or contributing cause of death. Nearly one in five died with pneumonia accompanied by contributing causes of chronic conditions, notably “old age.” Centenarians’ dying forms a picture of frailty exacerbated by the presence of a common stressor amongst older people of acute lung infection.

Patterns of cause of death changed with increasing age; this has implications in understanding differences in illness trajectories by age and has policy and service implications. The proportion of deaths from pneumonia increased with advancing age. In adults aged 70–84 years a comparatively small proportion were certified with pneumonia as the underlying cause of death (4.0%, 70–74 years [7]; 6.0%, 80–84 years). This number increased over 3-fold for centenarians (17.7%) and was commonly accompanied by increasing frailty and co-morbidities [32]. Centenarians’ experiences of living and dying with frailty are one of increased likelihood of “acute” decline from a stressor event accompanied by a background of frailty with declining physical function and vulnerability to a poor outcome following a stressor event, for example, an infection [33],[34].

An imperative for policy and services is the recognition of centenarians’ increased likelihood of “acute” decline and wider provision of advance care planning and anticipatory care with goals to promote quality of life and avoidance of crisis-driven interventions, notably hospital admission in the dying phase [35]. Wider recognition is required of the heterogeneous nature of illnesses’ trajectories that change with increasing longevity and service response to accommodate living with increasing frailty and vulnerability to acute decline. Wider recognition of centenarians’ high risk to a stressor event, notably pneumonia, amidst increasing chronic conditions [32], and frailty [37] could better tailor care provision that anticipates and plans for vulnerability to points of marked deterioration in health status and poor outcome. Illness trajectories for “frail” older people confer a marked deterioration in the last month of life [37],[38]. EoLC programmes and services need to anticipate and plan for the heterogeneity of decline experienced by frail elders [37], the intrinsic uncertainty as to how best to measure severity [34], and recognise when deterioration may precede the dying phase or be reversible. Studies report practitioners’ difficulties in recognising nearness to EoL for older people [39],[40]. A way forward is care not limited by prognostication, but directed by personal goals that seek to promote quality of life and anticipate frail elders’ vulnerability to “acute” deterioration in health status [34],[41].

Over the 10 years, trends in place of death little changed. More than one in four centenarians (27.2%) died in hospital. Most continued to die in a nursing or residential care home with little variation by cause of death. A small increasing proportion died at home. This finding follows patterns observed for people aged over 85 years who since 2006 have seen a reversal of a 30 year trend of declining death at home across all age groups[42], but this is mainly seen for people with cancer, not those with non-malignant conditions [43],[44].

The risk of dying in hospital for centenarians was associated with interplay between illness factors of cause of death, particularly pneumonia and ischaemic heart disease, and increasing contributing causes; individual factors, notably gender and environmental aspects, particularly higher level of deprivation and lower care home bed capacity. These findings support studies on place of death for older people that illustrate that gender, cause of death, socioeconomic status, and care home bed capacity have an important and complex effect on the likelihood of dying in hospital [5],[7],[19],[45]. Dying from cancer was associated with dying in one’s own home, but not with dying in a care home with or without nursing.

The rising number of centenarians and continued use of hospital care at the EoL indicates an urgent need to ensure adequate long-term care [20],[46]–[48] and responsive community care services to support people living with extreme longevity in these care settings. Compared to place of death for people aged 90 years or over in other European countries, the proportion dying in hospital in England is high and those dying in care homes low. For example, in the Netherlands and Finland most people aged over 90 years die in a long-term care setting (e.g., a nursing home) (90.6% [49] and 76.2% [50], respectively); few die in hospital (16.3% [51] and 13.6% [50], respectively). The increasing number of care home beds is positively associated with less likely death in hospital [49], but this alone is insufficient to explain the marked differences in place of death by age across European countries. Variations in health care service provision to care homes by country likely contribute to differences observed [25],[49],[52]. Better health care provision could enable people to remain in their usual residence and reduce hospital admission at the EoL—a major cost driver in EoLC [53].

Little variation between cause of death and dying in a care home with or without nursing has service implications, particularly for residential care homes that are social care settings reliant on primary health care services to meet residents’ health needs [26],[27]. The study’s findings suggest there is an equal if not greater need for EoLC in social care settings and adds weight to calls for improved EoLC in all settings. National EoLC interventions for care homes are mainly implemented in those with on site nursing. For example the Gold Standards Framework for Care Homes details EoLC interventions and staff training [54], although widely implemented with over 300 accredited care homes most are registered as a nursing home [55]. Moreover, most centenarians died from conditions rarely associated with the provision of specialist palliative care [56], with comparatively few dying from cancer and many from “old age.” These findings indicate centenarians’ reliance on general practitioners (a general physician) and community nurses to support EoLC provision, particularly in residential care homes. Gage and colleagues assert care home residents frequently experience a poor "fit" between their needs, and often *ad hoc* health care support hampered by limited strategic planning [26] and recognition of complex health needs associated with extreme longevity.

### Strengths and Limitations

Centenarians are a group often overlooked by policy makers and researchers. The study’s findings report analysis of a large unique dataset enabling detailed understanding on variations in cause of death by place of death for centenarians. The data comprise actual deaths over a specified time period for a specified group. The influence of variation in life expectancy on longevity is an area that requires further consideration to situate this work within, for example, the lower proportion of centenarian deaths in areas of greater deprivation, which reflects variance in life expectancy by region and level of deprivation.

Place of death formed the main outcome in the data analysis and associations, notably with cause of death. Although UK death certificate data are considered high quality [21], they do not encompass place of care or preferences for care in the period before death. The findings indicate associations with place of death, but prospective and longitudinal research is required to examine care and preferences in the preceding period to death. Certifying death is a complex medical process influenced notably by clinical uncertainty and family members of the deceased [57]. The many deaths certified as “old age” in community settings may relate both to diagnostic uncertainty with likely limited medical work up, or desirability for workup [37], and to protect the family with certification of death as “old age” as understandable and non-reversible. However, certifying death using ill-defined ICD-10 codes of malaise and fatigue (R53) or senility (R54) “old age” limits interpretation of cause of death and guidance of health strategies and programmes [21]. These ill-defined codes describe a symptom group rather than a defined disease [21]; a symptom group conceptualised as “frail” [33]. No associated causes of death are indicated for decedents where “old age” is the underlying cause of death. Underreporting of associated causes of death is likely. The ONS rule for death registration data dictates that if a contributing cause is stated this transposes “old age” as the underlying cause of death.

Future areas of research concern prospective cohort work on living and dying with advancing frailty to understand trajectories of disability in the last year of life to develop a conceptual model of living and dying with extreme longevity to inform health and social care policy. Replication of Mitchell and colleague’s [58] prospective cohort study of individuals with dementia to encompass “frailty” and extreme longevity is required.

In conclusion, dying in hospital from an “acute” cause or stressor event is common for centenarians in England. A policy imperative is the recognition of centenarians’ seemingly “hidden needs” of increased likelihood of “acute” decline and wider provision of anticipatory care to enable people to remain in their usual residence and reduce reliance on hospital care at the EoL. Increasing care home bed capacity could further reduce reliance on hospital care. The recognition of “acute” death amidst chronic contributing conditions illustrates the difficulties for people living and dying with extreme longevity. To better tailor care services requires prospective cohort work to examine the clinical course of extreme longevity and associated frailty.

## Supporting Information

Checklist S1 **STROBE Checklist.**
(PDF)

## Abbreviations

EoL: end of life
EoLC: end of life care
IMD: index of multiple deprivation indices
ONS: Office for National Statistics
PR: proportional ratio
