# Supplementary material for: Place and Cause of Death in Centenarians: A Population-Based Observational Study in England, 2001 to 2010
Source: PLoS Med. 2014 Jun 3;11(6):e1001653. doi: 10.1371/journal.pmed.1001653 (PMC4043499; doi:10.1371/journal.pmed.1001653)
Supplement: Checklist S1 — STROBE Checklist. (PDF) [file pmed.1001653.s001.pdf]

STROBE Statement—Checklist of items that should be included in reports of ***cross-sectional studies***

	Item No	Recommendation and response
**Title and abstract**	1	(*a*) Indicate the study's design with a commonly used term in the title or the abstract<br>**Title:** The hidden needs of centenarians dying in care homes: **a population-based observational study** in England
		(*b*) Provide in the abstract an informative and balanced summary of what was done and what was found<br>**Abstract methods:** A population-based observational study using death registration data linked with indices of multiple deprivation for people aged ≥100 years who died 2001to 2010 in England. Trends in number of deaths, place of death and cause of death were analysed by linear regression. Factors associated with place of death were evaluated using Poisson regression.
**Introduction**		
Background/rationale	2	Explain the scientific background and rationale for the investigation being reported<br>Centenarians are the fastest growing demographic group in the world. Yet, few studies consider the health and care needs of this group, how these differ from younger cohorts of older people or the implications for policy and service delivery of extreme longevity, notably end of life care. An important factor in informing end of life care is examination of cause of death data as a predictor of place of death.
Objectives	3	State specific objectives, including any prespecified hypotheses<br>This study aimed to examine trends and associated factors of place of death for centenarians in England over 10 years to inform health and social care policy and practice.
**Methods**		
Study design	4	Present key elements of study design early in the paper<br>A population-based observational study
Setting	5	Describe the setting, locations, and relevant dates, including periods of recruitment, exposure, follow-up, and data collection<br>Death registration data on all deaths from 2001-2010 in England
Participants	6	(*a*) Give the eligibility criteria, and the sources and methods of selection of participants. Describe methods of follow-up<br>Inclusion criteria comprised:<br>• Decedents aged 100 years or over at time of death<br>• Died in England between 2001 and 2010 (inclusive) from all causes of death, excluding external causes of accident or violence
		(*b*) For matched studies, give matching criteria and number of exposed and unexposed<br>Not applicable secondary analysis
Variables	7	Clearly define all outcomes, exposures, predictors, potential confounders, and effect modifiers. Give diagnostic criteria, if applicable<br>**Main outcome:** Place of death grouped into five categories: hospital, nursing home, residential care home, at home and elsewhere

		**Explanatory variables:** Detailed as three main groups: 1) demographic factors [age, gender and marital status, usual residence]; 2) illness related [ICD-10 codes for the top eight underlying causes of death and contributing causes of death]; and 3) environmental [deprivation, settlement type (e.g. rural, urban), number of care homes per 1,000 population].
Data sources/ measurement	8*	For each variable of interest, give sources of data and details of methods of assessment (measurement). Describe comparability of assessment methods if there is more than one group<br>Explanatory variables<br>1. Demographic factors: Individual level data derived from the Office for National Statistics (ONS) death registration data for England 2001-2010. The database details decedents' age, gender, marital status, usual residence, place of death and year of death<br>2. Illness related: Individual level data derived from the ONS death registration data for England 2001-2010 on underlying cause of death and contributing causes of death (up to 15) using *International Classification of Diseases Tenth Revision* (ICD-10)<br>3. Environmental variable data involved linkage of ONS death registration database with area level data on: deprivation, settlement type of place of residence, and care home bed capacity.<br>a. Deprivation using linkage with index of multiple deprivation indices (IMD) 2010 based on Lower Super Output Area (LSOA) of the decedents' usual residence s used regional level data on<br>b. Settlement type generated from decedent's usual residential address detailed in the ONS database.<br>c. Care home bed capacity derived from data linkage between ONS place of residence with data from the Care Quality Commission (CQC) (http://www.cqc.org.uk) to identify number of care home beds per 1000 population by decedents' local authority district.
Bias	9	Describe any efforts to address potential sources of bias<br>Inclusion all causes of death in the Poisson modelling by identifying first in descriptive analysis the main causes of death and grouping by ICD-10 codes. We identified and included seven disease groups that accounted for 90.2% of deaths with the remainder classified and included as 'other'. We checked residuals to test correct regression model specification.
Study size	10	Explain how the study size was arrived at<br>All deaths in England from 2001 to 2010 meeting the stated inclusion criteria
Quantitative variables	11	Explain how quantitative variables were handled in the analyses. If applicable, describe which groupings were chosen and why<br>The bivariate analysis finding informed candidate variables for the regression modelling on associations with place of death. Ten candidate variables identified and grouped as individual level data: i) demographic; ii) illness: and regional level data ii) environmental.
Statistical methods	12	(*a*) Describe all statistical methods, including those used to control for confounding

Data analysis

1. Basic descriptive analysis to describe demographic characteristics, causes of death, contributing causes and environmental factors (e.g. deprivation indices, died in usual residence). Causes of death were classified as: prominent specific disease types e.g. pneumonia ICD-10 J12-J118, disease group e.g. other respiratory ICD-10 J [others]; uncommon causes of death were collapsed into 'other' (those identified as outside the prominent ICD-10 categories).
2. Bivarirate analysis: prominent diseases/disease groups were entered into the bivariate analysis using frequency tables and descriptive statistics (e.g. Chi^2 categorical data, non-parametric test for ordinal data) to explore place of death and variation by gender, marital status, causes of death, number of contributing causes, deprivation, region and settlement (urban/rural).
3. Poisson regression: the bivariate analysis finding informed candidate variables for regression modelling on associations with place of death with 10 candidate variables grouped as individual level data: i) demographic; ii) illness: and regional level data ii) environmental. Multivariable Poisson regression with robust error variance to calculate proportional ratios, to investigate factors associated with hospital death versus care home (nursing home or residential home) or home. Age and year of death remained in the model as continuous variables. We checked residuals to test correct model specification.

(*b*) Describe any methods used to examine subgroups and interactions

- Bivarirate analysis of prominent diseases/disease groups using frequency tables and descriptive statistics to explore place of death and variation by gender, marital status, causes of death, number of contributing causes, deprivation, region and settlement (urban/rural). Analysis informed candidate variables included in the Poisson regression.
- Poisson regression on associations with place of death as hospital as reference verses residential care home, nursing home or own residence with 10 candidate variables grouped as individual level data: i) demographic; ii) illness: and regional level data ii) environmental.

(*c*) Explain how missing data were addressed

Key variables were checked within the data set and decedents excluded if missing. Outcome, complete data set. No decedents excluded because of missing data.

(*d*) If applicable, explain how loss to follow-up was addressed

(*e*) Describe any sensitivity analyses

Results

Participants 13* (a) Report numbers of individuals at each stage of study—eg numbers potentially eligible, examined for eligibility, confirmed eligible, included in the study, completing follow-up, and analysed

The sample comprised 35,867 people. This included all deaths for people aged 100 years or over in England from 2001-2010. Excluded those who died

		from external causes.
		(b) Give reasons for non-participation at each stage<br>Not applicable death registration data
		(c) Consider use of a flow diagram
Descriptive data	14*	(a) Give characteristics of study participants (eg demographic, clinical, social) and information on exposures and potential confounders<br>The sample comprised 35,867 people with an mean age of 101.4 (sd 1.7; median 101, range: 100-115 years) at time of death, mainly women (86.7%) and widowed (85.0%). Areas of highest deprivation had the lowest proportion of centenarian deaths with consequent regional variance by level of deprivation. The North East of England had the lowest proportion of centenarian deaths (4.3%).
		(b) Indicate number of participants with missing data for each variable of interest<br>Accounted for <1% of the sample
		(c) Summarise follow-up time (eg, average and total amount)<br>Not applicable death registration data
Outcome data	15*	Report numbers of outcome events or summary measures over time<br>Main outcome place of death categorised as: hospital, care home with nursing, care home without nursing, own residence or elsewhere. Hospital formed the reference group in the Poisson regression verses care home with nursing or care home without nursing or own residence.
Main results	16	(*a*) Give unadjusted estimates and, if applicable, confounder-adjusted estimates and their precision (eg, 95% confidence interval). Make clear which confounders were adjusted for and why they were included<br>We used Poisson regression with robust error variance to calculate proportional ratios, to investigate factors associated with hospital death versus care home (nursing home or residential home) or home. Age and year of death remained in the model as continuous variables. We used 95% confidence intervals.
		(*b*) Report category boundaries when continuous variables were categorized<br>Category boundaries for continuous variables included:<br>• Number of contributing causes of death (up to 15 stated on death certificate) categorised as: 0, 1, 2, 3 or 4+
		(*c*) If relevant, consider translating estimates of relative risk into absolute risk for a meaningful time period<br>Not applicable
Other analyses	17	Report other analyses done—eg analyses of subgroups and interactions, and sensitivity analyses<br>**Factors associated with place of death (reference: hospital)**<br>**Demographic factors**<br>Being a woman increased the likelihood of dying outside of hospital in a community setting of a care home (with or without nursing) and own residence (table 3). Influence of marital status was equivocal (table 3).

		**Illness factors**<br>Underlying causes of death (reference: dementia) were associated with place of death (table 3, p<0.001). People with an underlying cause of death from cancer (PR 0.86,95%CI 0.79-0.92), ischaemic heart disease (PR 0.92,95%CI 0.86-0.97), or other circulatory diseases (PR 0.93,95%CI 0.88-0.99) were less likely to die in hospital than at home. There was little variance between cause of death and dying in a care home with nursing or without (table 3). Only 'old age' as a cause of death was associated with being less likely to die in hospital compared with a nursing home (PR 0.78,95%CI:0.69-0.87) or residential care home (PR 0.58,95%CI: 0.52-0.65,p<0.001)). Dying with a more cumulative picture of disease with ≥ 4 contributing causes of death was associated with dying in hospital not care home either with nursing (PR 1.27,95%CI:1.21-1.33) or without (PR 1.36, 95%CI:1.29-1.43). Conversely, those dying with a single contributing cause were less likely to die in hospital and more likely to die in a care home with nursing (PR 0.84, 95%CI:0.81-0.87) or without (PR 0.80,95%CI:0.77-0.84) or own residence (PR 0.91, 95%CI:0.88-0.93).<br>**Environmental factors**<br>Higher numbers of care home beds were associated with fewer hospital deaths and more deaths in care homes (with nursing PR 0.98,95%CI: 0.98-0.99, p<0.001; or without nursing PR 0.98,95%CI: 0.97-0.98, p<0.001). The main difference between dying in hospital and care home type pertained to a higher prevalence of dementia in nursing homes (21.3% v 16.5%) (table 3). Once removed from the model minimal differences were observed between cause of death and care home type (Chi^2 13.98, df=7 p=0.051).<br>Levels of deprivation and settlement type influenced place of death. Areas most deprived showed greatest association with dying in hospital not in a community setting (table 3). Dying outside of hospital in own residence related to usual residence of urban versus rural settlement (PR 0.85,95%CI: 0.81-0.89) or town/fringe (PR 0.95, 95%CI: 0.92-0.99).
**Discussion**		
Key results	18	Summarise key results with reference to study objectives<br>Over the 10 years, trends in place of death little changed with most centenarians dying in a nursing or residential care home with little variation by cause of death. However, more than one in four centenarians (27.2%) died in hospital. Dying in hospital rather than in a care home was associated with cause of death, increasing contributing causes of death, being male and care home bed capacity. Wider recognition of centenarians' seemingly 'hidden needs' of increased likelihood of 'acute' decline is necessitated. 'Acute' causes of death were common with nearly one in five dying from pneumonia accompanied by contributing causes of chronic conditions, particularly 'old age'. A small, but increasing proportion of centenarians died at home.
Limitations	19	Discuss limitations of the study, taking into account sources of potential bias or imprecision. Discuss both direction and magnitude of any potential bias<br>The study's findings report analysis of a large unique data set enabling detailed understanding on variations in cause of death by place of death for centenarians; a group often overlooked by policy makers and researchers. The findings are limited by the accuracy of the primary data source; death certificates. Place of death formed the main outcome in the data analysis. Although this data are considered high quality, they do not encompass place

		of care or preferences for care in the period before death. The findings examine associations with place of death, but prospective and longitudinal research is required to examine care and ideally preferences in the preceding period to death.<br><br>Certifying death using ill-defined ICD-10 codes such as malaise and fatigue (R53) or senility (R54) 'old age' limits interpretation of cause of death and guidance of health strategies and programmes. Although infrequently used in UK death certificates, ICD-10 codes for 'old age' accounted for 28.1% of the decedents' cause of death. These ill-defined codes describe a symptom group rather than a defined disease; a symptom group conceptualised as 'frail'. No associated causes of death are indicated for decedents where 'old age' is the underlying cause of death. Underreporting of associated causes of death is likely. The ONS rule for death registration data dictates that if a contributing cause is stated this transposes 'old age' as the underlying cause of death. Overestimates of dying outside usual residence may occur where an informant gave a private address for a decedent who died in care home where they had lived for many months
Interpretation	20	Give a cautious overall interpretation of results considering objectives, limitations, multiplicity of analyses, results from similar studies, and other relevant evidence<br><br>Centenarians die mainly in care homes with or without nursing care; a significant unchanging proportion continue to die in hospital. The recognition of 'acute' death, notably form pneumonia, amidst chronic contributing conditions and frailty illustrates the difficulties for people living and dying with extreme longevity. The findings show a policy imperative is the recognition of centenarians' seemingly 'hidden needs' of increased likelihood of 'acute' decline and wider provision of anticipatory care. To better tailor care services requires prospective cohort work to examine the clinical course of extreme longevity and associated frailty.
Generalisability	21	Discuss the generalisability (external validity) of the study results<br>The study's findings report analysis of a large unique data set comprising all centenarian deaths in England from 2001 to 2010 enabling detailed understanding on variations in cause of death by place of death for centenarians; a group often overlooked by policy makers and researchers. The reporting of 95% confidence intervals enables consideration of the findings in relation to the wider population outside of England.
**Other information**		
Funding	22	Give the source of funding and the role of the funders for the present study and, if applicable, for the original study on which the present article is based<br><br>The GUIDE_Care project was funded by the National Institute for Health Research Health Services and Delivery Research(NIHR HS&DR) programme(Project number 09/2000/58). Dr Catherine Evans is funded by a NIHR/Chief Nursing Officer Clinical Lectureship award and sponsorship from Sussex Community NHS Trust. Professor Irene Higginson is an NIHR Senior Investigator.

*Give information separately for exposed and unexposed groups.

Note: An Explanation and Elaboration article discusses each checklist item and gives methodological background and published examples of transparent reporting. The STROBE checklist is best used in conjunction with this article (freely available on the Web sites of PLoS Medicine at http://www.plosmedicine.org/, Annals of Internal Medicine at http://www.annals.org/, and Epidemiology at http://www.epidem.com/). Information on the STROBE Initiative is available at http://www.strobe-statement.org.
